# The rare and the common: habitat suitability and niche segregation of *Anopheles algeriensis* (Theobald, 1903) and *Anopheles claviger* s.s. (Meigen, 1804) in North-German peatlands is shaped by distinct landscape–weather interactions

**DOI:** 10.1186/s13071-026-07612-w

**Published:** 2026-09-23

**Authors:** Patrick Gutjahr, Amelie Weber, Susanne Fischer, Jürgen Kreyling, Franziska Tanneberger, Mandy Schäfer

**Affiliations:** 1 Friedrich-Loeffler-Institut – Federal Research Institute for Animal Health (FLI), Institute of Infectology, Greifswald, Insel Riems Germany; 2 Greifswald Mire Centre, Institute of Botany and Landscape Ecology, Greifswald University, Greifswald, Germany

**Keywords:** *Anopheles algeriensis*, *Anopheles claviger* s.s., Mosquito-borne disease, One Health, Peatland, Landscape ecology, Rewetting, GLMM

## Abstract

**Background:**

Peatland rewetting is a cost-effective strategy for climate change mitigation and biodiversity conservation, yet its potential to alter mosquito species composition and abundance carries implications for public and veterinary health. *Anopheles algeriensis* and *Anopheles claviger* s.s. are broadly distributed across European wetlands, including peatlands, but despite their vector potential, their specific ecological niches and responses to rewetting remain poorly understood.

**Methods:**

Adult mosquitoes were sampled biweekly across two consecutive seasons at 15 sites within a fen peatland landscape in North-East Germany and identified morphologically. Anopheline specimens outside the *Anopheles maculipennis* complex underwent further molecular identification. Sampling sites were grouped into three habitat types, wet peatlands, drained peatlands, and settlements, based on key peatland characteristics. Subsequently, generalised linear mixed models were used to assess species-specific responses to habitat type and their interactions with relevant weather variables.

**Results:**

A total of 278 *An. algeriensis* individuals were collected, with significantly higher abundances observed in wet peatlands than in other habitat types. Mean temperature and mean relative humidity were positively correlated with abundance. The former independent of habitat types, the latter was modulated by them. Cumulative precipitation was negatively correlated with abundance, varying in strength across habitat types. In contrast, 950 *An. claviger* s.s. specimens were most abundant in settlements. Mean relative humidity was positively correlated with abundance in settlements, but negatively in other habitat types. Minimum temperature during trap nights was positively correlated with abundance in settlements, and in wet peatlands, but negatively in drained peatlands.

**Conclusions:**

Distinct distribution and abundance patterns driven by landscape–weather interaction were identified for two *Anopheles* vectors. Our findings demonstrate species-specific responses to peatland habitat types and highlight the need to integrate vector surveillance into rewetting and management strategies within a One Health framework.

**Graphical Abstract:**

**Figure:**
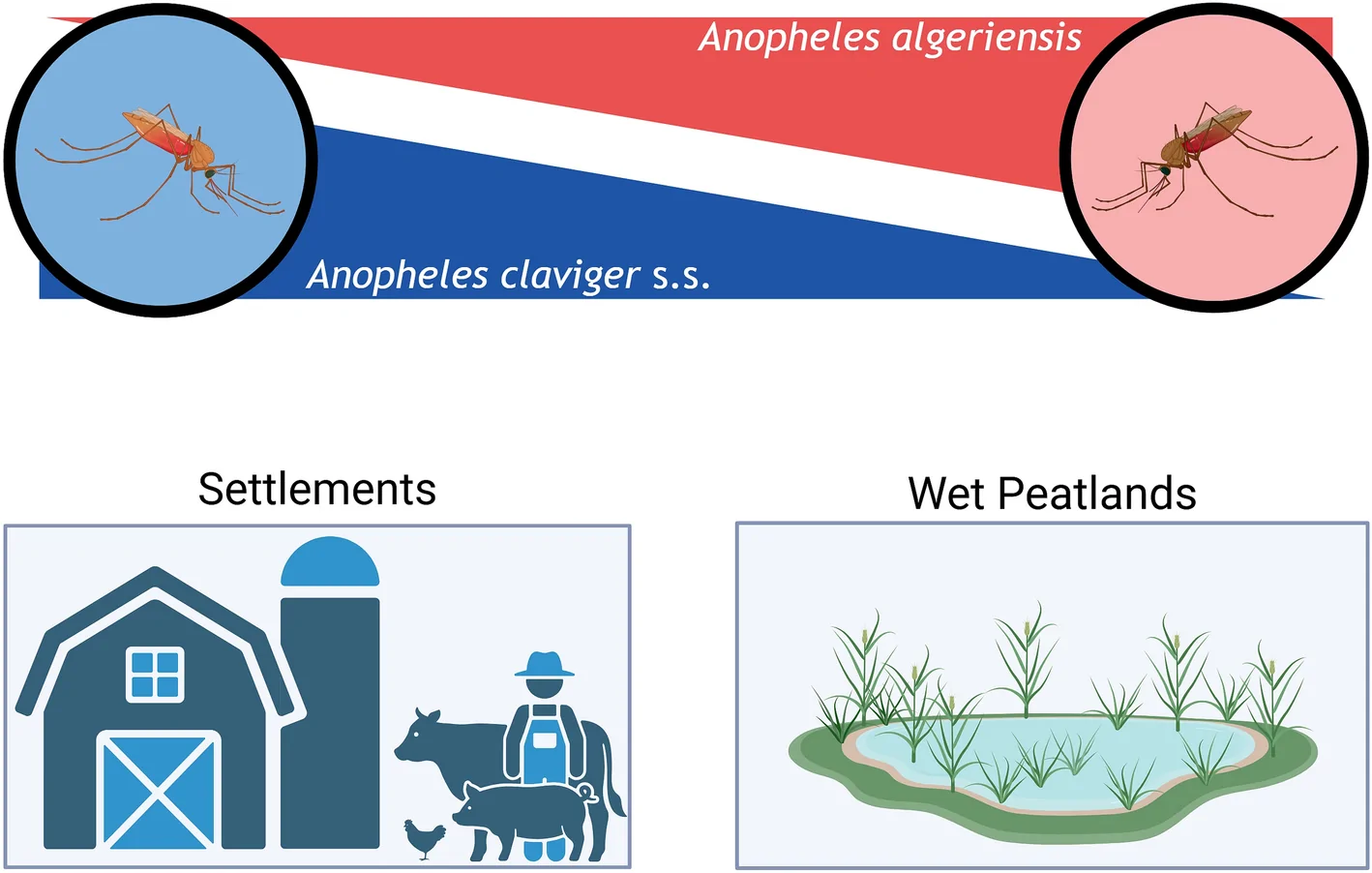

**Supplementary Information:**

The online version contains supplementary material available at https://doi.org/10.1186/s13071-026-07612-w.

## Background

Peatlands provide essential ecosystem services including climate and water regulation or habitat for specialised and endangered species [1]. Their unique trait among ecosystems is the vast amount of carbon stored in their peat [2]. Peatlands can be divided into bogs, those exclusively supplied by precipitation, and fens, which are also fed by ground and/or surface water [3]. While globally the majority of bogs and fens remain in undrained condition, this is very different in Europe [1]: for example, over 90% of German peatlands have been drained, turning them from carbon sinks into sources, accounting for approximately 7% of the country's annual greenhouse gas (GHG) emissions [4]. Peatland rewetting restores high water tables and peat-forming vegetation leading to immediate emission reduction. Thus, it is a core strategy in climate mitigation and biodiversity restoration [5]. However, re-establishing water-saturated soils may also re-create or re-expand mosquito habitats, including those suitable for anopheline species, with potential consequences for ecosystem dynamics or public and veterinary health [6, 7].

*Anopheles* mosquitoes (Diptera: Culicidae), the sole vectors of human malaria, have shaped human history and remain of epidemiological concern even in regions where the disease has been eradicated [8–10]. Malaria was historically endemic across Europe, including Germany [11] and its elimination has been attributed in part to reduced breeding site availability following peatland drainage [12]. Although the current risk of autochthonous transmission is considered negligible, competent vector species remain widespread while international travel and migration continue to introduce *Plasmodium* spp. [13–15]. Additionally, arboviruses and filarial nematodes are frequently detected in *Anopheles* species [16, 17]. As mosquito-borne diseases are projected to increase [18], surveillance of anopheline mosquitoes is particularly critical in the context of wetland restoration.

Nine *Anopheles* species occur in Germany, differing in ecology, spatiotemporal distribution, and vector competence [19, 20]. The vector potential of the most recent addition, *An. hyrcanus* is yet undetermined but its rapid northward expansion illustrates the ongoing shifts in European mosquito distributions [20]. Historically, the endemic members of the *An. maculipennis* complex (*An. atroparvus*, *An. messeae*, *An. daciae* and *An. maculipennis* s.s*.*) were the primary vectors of human-pathogenic *Plasmodium* spp. in Germany. Consequently, the environmental factors driving their distribution have been studied thoroughly [21, 22]. The secondary vectors include *An. algeriensis, An. plumbeus* and the *An. claviger* complex, comprising the nominative species *An. claviger* s.s. and its sibling *An. petragnani* [19]. Thus far, only the habitat preference of *An. plumbeus* have been described in detail [23]. *Anopheles petragnani* was detected in Germany only a decade ago and is morphologically indistinguishable from its sibling species, making regional distribution and ecological requirements difficult to assess [24]. By contrast, *An. claviger* s.s. and *An. algeriensis* are documented in a range of habitats yet differ markedly in ecology, adaptability, and by extension in epidemiological relevance [25]. *Anopheles algeriensis* is mostly found around the Mediterranean, but has been documented across much of Europe [26], with records extending to the UK [27], the Netherlands [28], Germany [29], Sweden [30], and Estonia [31]. Despite this wide distribution, it is regarded as one of the rarest mosquito species in Europe [32]. It shows little tolerance for anthropogenic environments and is typically reported from wetlands including marshes and reed beds [29, 32, 33], a vegetation structure often dominant in rewetted fen peatlands [34].

Conversely, *An. claviger* s.s. inhabits a broad spectrum of aquatic environments including natural and artificial water bodies, demonstrating greater ecological flexibility and wider distribution across Germany and Europe [25, 27]. It is commonly recorded in mosquito monitoring programmes, often in high abundance [19]. Occasional sympatric occurrence with *An. algeriensis* further complicates the delineation of habitat requirements unique to each species [33]. Recent research indicates geochemical parameters, such as pH and salinity, vary among breeding sites [35], parameters which are altered by rewetting [34].

To further unravel niche specific characteristics, we analysed species distribution patterns of adult *An. claviger* s.s. and *An. algeriensis* in a fen peatland landscape in North-East Germany. The local-scale of the study, extending approximately 8 km along the east–west axis and 4 km along the north–south axis, justified two key assumptions. First, weather patterns were uniform across the study area, ensuring consistent environmental drivers of mosquito abundance [36]. Second, sampled specimens likely represented single populations per species, since ecological preferences and responses can vary among conspecific populations [37–39]. This allowed assessment of ecological responses without confounding effects of the genetic variation described for *An. claviger* complex [40] and *An. algeriensis* [41] across Europe. Given the rare occurrence of *An. algeriensis*, cryptic species within the *An. claviger* complex and the landscape diversity on site, we employed genetic and statistical methods to: (1) determine the species composition, (2) assess species-specific landscape associations, and (3) characterise landscape–weather interactions shaping ecological niches of *An. algeriensis* and *An. claviger* s.s. to facilitate evidence-based peatland management aligned with One Health principles [42].

## Methods

### Study area

**Fig. 1:**
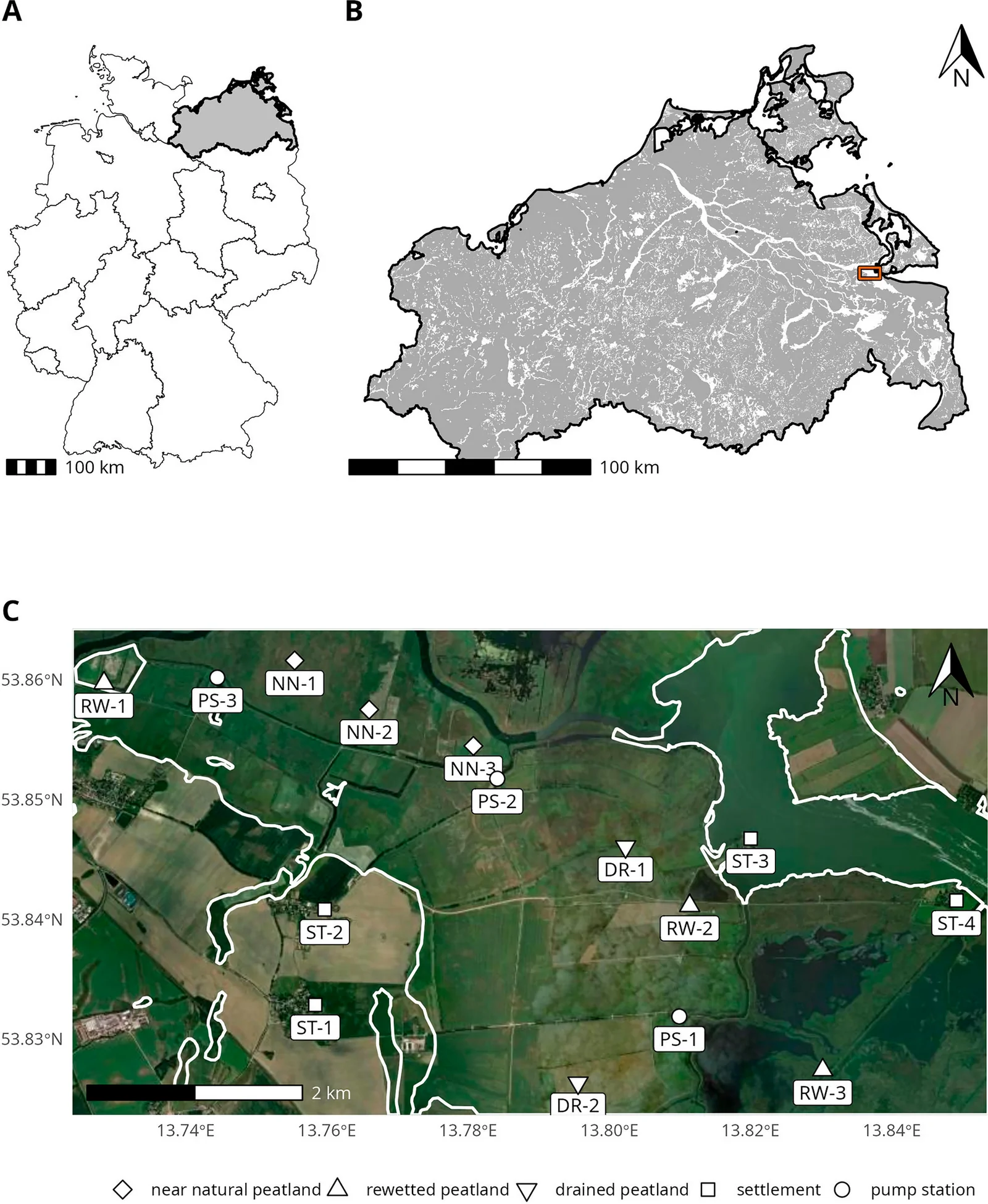
**A** Map of Germany with Mecklenburg-Vorpommern indicated by grey shading. **B** Map of Mecklenburg-Vorpommern showing peat soils as white areas [54], with the area of interest (AOI) marked by an orange square at the central eastern coast of the state. **C** Satellite image of the AOI showing the sampling sites, including their locations, names, and corresponding landscape structure. The area outlined in white indicates the extent of peat soils [54]

The study was conducted in the Lower Peene Valley, a lowland fen landscape in North-East Germany (Fig. 1A, B). The region exhibits a mosaic of rewetting stages, vegetation types and land-use patterns making it particularly suitable to investigate mosquito diversity in peatlands. To account for structural landscape complexity, the sampling design considered three sites with near-natural hydrology (NN1–3), three rewetted sites (RW1–3), two drained peatland sites (DR1–2) and four settlement sites (ST1–4) on mineral soil adjacent to the peatland (Fig. 1C). In addition, to identify possible edge effects between the drained and wet peatland areas, the catch basins of three pump stations (PS1–3) were also included. The total area of interest (AOI) is 3,853 ha (Fig. 1C). The satellite imagery was provided by ESRI and accessed via the *basemaps* package. All visualisation, data transformation, and analyses in this study were conducted in R Statistical Software (Version 4.5.3) and RStudio IDE (Version 2026.01.2 + 418) [43–45]. Detailed site descriptions are provided in Additional file 1.

### Mosquito sampling and identification

Adult mosquito populations were monitored biweekly over 24-h periods from April to October in 2023 and 2024. The sites were sufficiently far apart to minimise interference (range: 377 m–8195 m, mean: 3316 m, median: 2926 m) [46]. Sampling was conducted using BG-Pro traps (Biogents, Germany) in CDC configuration, positioned with shutters 1–1.5 m above ground level. Each trap was supplemented with BG-Mozzibait olfactory attractant (Biogents, Germany) and CO₂ from 10 kg cylinders. The flow rate was calibrated to 20.8 g/h prior to each trap night using a red-y Portable Mass Flow Meter (Vögtlin, Switzerland), corresponding to the manufacturers recommended 500 g/d [47].

Following collection, specimens were transported to the laboratory and preserved at -20 °C until processing. Morphological identification was carried out utilising taxonomic keys by Becker et al. 2020 [25], Cranston et al. 1987 [48] and Lindström & Eklöf 2022 [49]. Identified specimens were individually stored in 2-ml reaction tubes and maintained at -80 °C for long-term preservation, each with unique IDs to document taxa, sex, collection site, and date. Males were only identified to genus level and excluded from further analysis.

### Molecular species identification and pathogen screening

To ensure accurate identification of damaged specimens or species complex members, all *Anopheles* specimens not morphologically assigned to the *An. maculipennis* complex underwent molecular analysis. Individual specimens were homogenised by adding two 3 mm steel beads and 3 min disruption at 30 Hz using a TissueLyser II bead mill (Qiagen, Germany), and nucleic acids were extracted with the NucleoMag Vet kit (Macherey–Nagel, Germany) on a KingFisher Flex platform (ThermoFisher Scientific, USA). Then all specimens underwent an *ITS2*-targeting PCR assay differentiating members of *An. claviger* complex [50]. Non-amplifying specimens were subsequently identified by *COI* barcode sequencing [51]. All PCR amplifications used the QuantiTect Multiplex PCR NoROX Kit (Qiagen, Germany). Cycle sequencing was performed with the BigDye Terminator v3.1 kit (ThermoFisher Scientific, USA), cleaned with NucleoSEQ columns (Macherey–Nagel, Germany), and analysed on an ABI 3730 capillary sequencer (ThermoFisher Scientific, USA). Sequences were edited and aligned against GenBank reference sequences in Geneious Prime (v2025.2.2).

Additionally, all specimens were screened to detect members of the superfamily Filaroidea using a SYBR Green real-time PCR assay targeting the mitochondrial *12S* rRNA gene [17], run on a CFX96 Detection System (Bio-Rad, USA) with the QuantiTect SYBR Green PCR Kit (Qiagen, Germany). All analysis followed the manufacturer protocols and included positive and negative controls.

### Environmental data collection

To account for broader landscape composition, the digital landscape model (DLM) surrounding the traps was reclassified into three principal landscape characteristics [52]: (A) predominant vegetation, (B) soil status including moisture classes for peat soil based on representative water table measurements, and (C) land-use intensity based on agricultural practices (maps created with the *ggspatial* package [53] provided in Additional file 2, Figs. 1A–C). The DLM is publicly available through the State Office for Internal Administration Mecklenburg-Vorpommern (https://www.laiv-mv.de/, target scale: 1:25,000, point accuracy: ± 3 m), peat soil extent is based on Wittnebel et al. [54].

To account for landscape effects at relevant scales, two buffer radii were defined around each trap. One radius (400 m, approx. 50 ha) was defined based on the average non-oriented flight of mosquitoes in general reported in a meta-analysis and concurring with the upper threshold at which landscape anthropisation affects mosquito communities since no representative species-specific data were available [55, 56]. The other radius (5.6 m, approx. 100 m^2^) reflected fine-scale habitat heterogeneity of the immediate trap surroundings. For both radii, relative feature composition across the three principal landscape characteristics was calculated using the *sf* package [57, 58], yielding 42 variables per site (seven features per landscape characteristic, three characteristics across two radii). Administratively defined polygons were excluded because they lack biological relevance, as were point and line features due to their lack of spatial extent. Standing and flowing water were retained from the DLM. Although all standing water within the AOI overlies peat soils and is unmanaged, it was kept as a distinct category because breeding and resting site availability may differ from adjacent areas, particularly in relation to oviposition behaviour [59, 60]. Accordingly, standing and flowing water were integrated across all characteristics. To improve interpretability and avoid data gaps or disproportionate weighting, sealed surfaces and buildings were combined into a single settlement category, which was likewise included across all characteristics. Subsequently, the *vegan* package was employed to compute a Euclidean dissimilarity matrix and perform hierarchical clustering using Ward’s minimum variance method (Ward.D2) [61]. Visual inspection yielded three meaningful habitat types which were referred to as “wet” (peatlands), “drained” (peatlands) and “settlements” (Additional file 2, Fig. 2).

Meteorological data were obtained from the nearest German Weather Service (DWD) station in Anklam (Station ID: 167), located 3–10 km west of the sampling sites (https://opendata.dwd.de/) [62]. Colour palettes for all visualisations were selected using the *col4all* package in accordance with recommendations [63, 64].

### Statistical analysis

Following the descriptive overview, distribution and abundance of both vector species were compared among the predefined categories (Fig. 1C) versus the more objective derived habitat types, using Kruskal–Wallis tests (*rstatix* package) on individual trap nights, followed by Dunn's post hoc pairwise comparisons with Bonferroni-adjusted p-values. [65]. As results were largely consistent between the two schemes, subsequent analyses proceeded with the more objective habitat types. A negative binomial distribution was then identified via Cullen and Frey graphs (*fitdistrplus* package; Additional file 2, Figs. 3–4) [66]. To further investigate distribution and abundance patterns of both species, along with potential interactions between climate variables and habitat type, generalised linear mixed models (GLMMs) with a log link function were fitted. Fixed effects comprised cumulative 14-day precipitation (pt14), mean 14-day temperature (dtm14) and relative humidity (rhm14), as well as minimum temperature during trap night (temp_min), variables repeatedly shown to reliably characterise weather-dependent mosquito dynamics [36].

Week-by-year interactions and sampling location were included as random effects in all models to account for temporal, spatial, and spatiotemporal dependencies. Starting from a maximal model, comprising all main effects plus habitat types interacting with climate variables, and a base model, consisting of main effects only, backward selection was performed by sequentially removing non-significant terms and comparing nested models via AIC, BIC, and log-likelihood (*glmmTMB* package) [67, 68]. As the a priori hypothesis concerned whether abundance was modulated by climatic variables in a habitat type specific manner, rather than joint effects among the climatic variables themselves, interactions among climatic variables were excluded from the models. Model optimisation involved residual diagnostics and assessment of spatial and temporal autocorrelation using the *DHARMa* package [69]. Model evaluation and post hoc analyses involved Type III analysis of variance (ANOVA, *car* package), collinearity among fixed effect predictors was assessed using the *performance* package. [70, 71]. To enhance interpretability and facilitate visualisation, model results were evaluated using the *emmeans* and *sjPlot* packages [72, 73].

## Results

### Mosquito sampling, identification and pathogen screening

Between 28 April 2023 and 14 October 2024, 26 trap nights were conducted at all 15 sites, yielding 390 sampling events. Data from 10 to 11 July 2024 were excluded due to storm-related technical interference. Of 1230 anopheline specimens not belonging to the *An. maculipennis* complex, two were *An. plumbeus* (ST 1, 29 June 2023; RW 2, 22 August 2024) and not analysed further. Of the remainder, 278 were identified as *An. algeriensis* and 950 as *An. claviger* s.s*.* Neither *An. petragnani* nor filarial nematodes were detected. *CO1* barcodes of both *An. plumbeus* specimens and two randomly selected representatives each of *An. claviger* s.s. and *An. algeriensis* were submitted to NCBI GenBank (accession numbers PZ177929 to PZ177934).

### Descriptive statistics

**Fig. 2:**
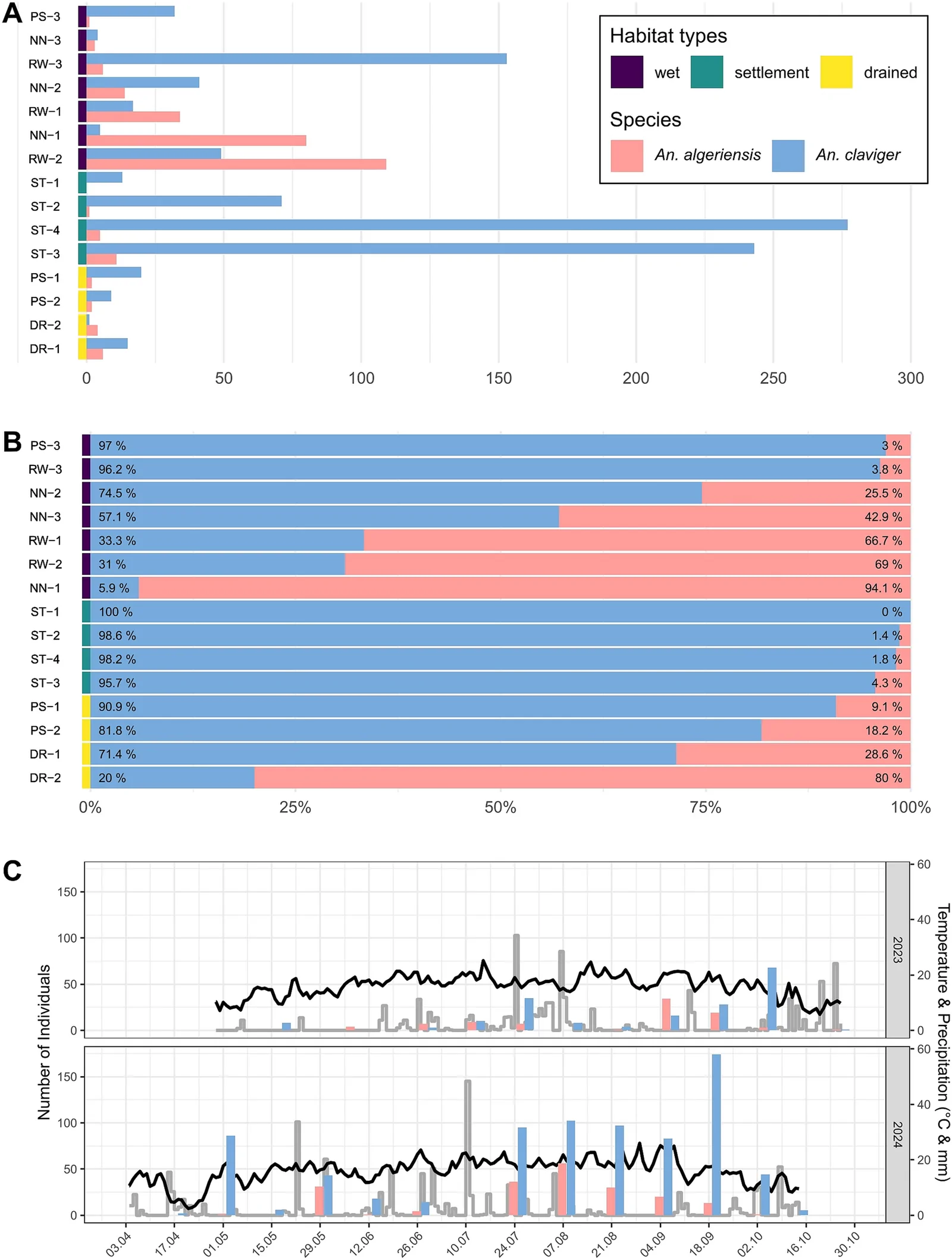
**A** Cumulative and **B** relative distribution of both *An. claviger* s.s. and *An. algeriensis* at all 15 sampling sites. The respective habitat type is indicated by the coloured bar next to the site labels. **C** Annual phenology of both *An. claviger* s.s. and *An. algeriensis* cumulative per 24 h trap night at all 15 sampling sites. The grey stepped line indicates cumulative precipitation per day, the black line indicates daily mean temperature

The cumulative distribution of both species (Fig. 2A) shows *An. claviger* s.s. more abundant at 11 of 15 sites (73.3%), ranging from 1 specimen (20%) at DR 2 to 277 (98.2%) at ST 4, whereas *An. algeriensis* was absent at ST 1 but reached 109 specimens (69%) at RW 2. Relative abundance (Fig. 2B) shows *An. claviger* s.s. dominated settlement sites (min = 95.7%, max = 100%, mean = 98.1%), whereas *An. algeriensis* was more heterogeneous, peaking in wet peatland (min = 3%, max = 94.1%, mean = 43.6%) and drained peatland (min = 9.1%, max = 80%, mean = 34%), the latter at lower overall abundance. Annual phenology (Fig. 2C) revealed two population peaks for both species, in late spring and late summer, with the latter more pronounced. The most frequent sympatric species with *An. claviger* s.s. were *Culex pipiens/torrentium* (n = 9645), while *An. algeriensis* most frequently co-occurred with *Ae. cinereus/geminus* (*n* = 8476). Catch data are provided in Additional file 4.

### Model results for* Anopheles claviger* s.s.

Abundance of *An. claviger* s.s. differed significantly among habitat types (Kruskal–Wallis: *H* = 23.3, *df* = 2, *P* < 0.0001). Median abundance was significantly higher in settlements (median = 157, total = 604) than in wet peatlands (median = 32, total = 301, *P* < 0.0001) and drained peatlands (median = 12, total = 45, *P* = 0.0001; Bonferroni-adjusted Dunn's test), while the two peatland types did not differ significantly (*P* = 1; details in Additional File 3, tables T1 & T2).

**Fig. 3:**
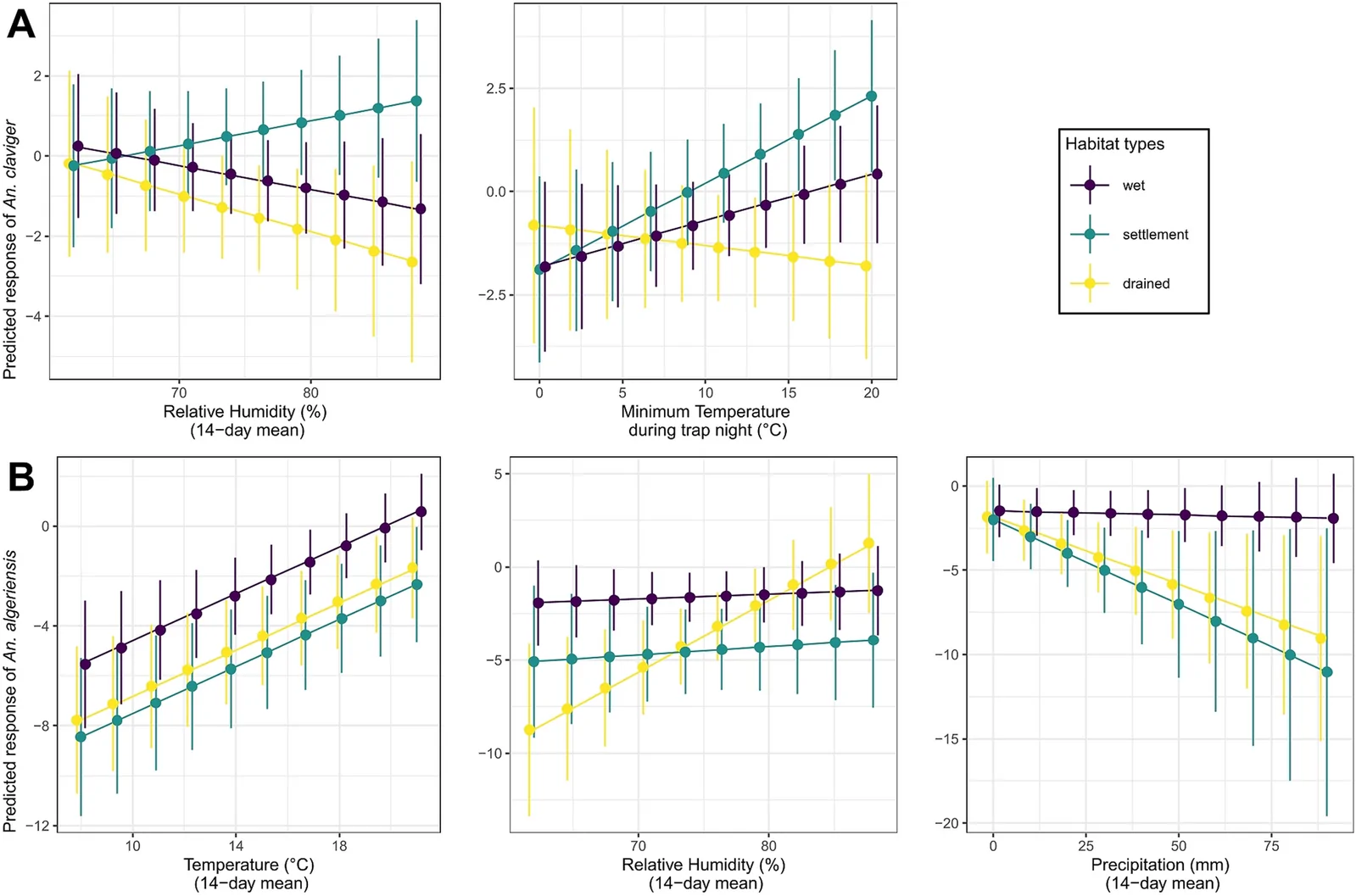
GLMM results predicted for ten equidistant values within measurement range for the retained climate effects. For **A** *An. claviger* s.s. the two retained effects are “rhm14: 14-day mean relative humidity” (left) and “temp_min: minimum temperature during trap night” (right). For **B** *An. algeriensis* the three retained climate effects are “dtm14: 14-day mean daily temperature” (left), “rhm14: 14-day mean relative humidity” (centre) and “pt14: 14-day cumulative sum of precipitation” (right). The graphs are on the log scale, colours indicate habitat types. 95% confidence intervals are represented by vertical lines per predicted value

After model optimisation, with the number of *An. claviger* s.s. per sampling event as the response variable, minimum trap-night temperature and relative humidity were retained as predictors, both interacting with habitat type. No autocorrelation or relevant multicollinearity was detected. Coefficients were examined relative to drained peatlands as the reference category. Minimum temperature showed no significant main effect (*β* =  − 0.18, SE = 0.42, *P* = 0.66), but a significant positive interaction in settlements (*β* = 0.96, SE = 0.39, *P* = 0.01) while the interaction in wet peatlands was not significant (*β* = 0.60, SE = 0.38, *P* = 0.12). Relative humidity likewise showed no significant main effect (*β* =  − 0.53, SE = 0.45, *P* = 0.24), but interacted significantly and positively with settlements (*β* = 0.87, SE = 0.43, *P* = 0.04), but not with wet peatlands (*β* = 0.19, SE = 0.41, *P* = 0.64). Settlements showed significantly higher abundance than the reference (*β* = 1.94, SE = 0.80, *P* = 0.02), whereas wet peatlands did not differ significantly (*β* = 0.87, SE = 0.72, *P* = 0.23; post hoc results in Additional File 2, page 4). Marginal trends (Fig. 3A) showed that relative humidity effects varied in direction across habitats: positive in settlements (*β* = 0.06, 95% CI [− 0.06, 0.19]) and negative in both, drained (*β* =  − 0.09, 95% CI [− 0.25, 0.06]) and wet peatlands (*β* =  − 0.06, 95% CI [− 0.18, 0.06]). Minimum temperature effects also diverged: positive in settlements (*β* = 0.21, 95% CI [0.04, 0.38]) and wet peatlands (*β* = 0.11, 95% CI [− 0.05, 0.27]), but negative in drained peatlands (*β* =  − 0.05, 95% CI [− 0.27, 0.17]). The transformed incidence rate ratios (IRRs) for all predictors and interactions, along with significance levels, are provided in Additional file 2, Fig. 5A.

### Model results for *Anopheles algeriensis*

Abundance of *An. algeriensis* also differed significantly among habitat types (Kruskal–Wallis: H = 17.0, df = 2, *P* = 0.0002), but the pattern differed from *An. claviger* s.s. as well: median abundance was significantly higher in wet peatlands (median = 14, total = 247) than in drained peatlands (median = 3, total = 14; *P* = 0.002) and settlements (median = 3, total = 17; *P* = 0.002; Bonferroni-adjusted Dunn's test), while drained peatlands and settlements did not differ significantly (*P* = 1; details in Additional File 3, T1 & T2).

In the optimised model, with the number of *An. algeriensis* per sampling event as the response variable, mean daily temperature, relative humidity, and cumulative precipitation were retained as predictors, the latter two interacting with habitat type (drained peatlands as reference). Neither autocorrelation nor problematic multicollinearity was detected. Mean daily temperature showed a strong positive main effect (*β* = 1.63, SE = 0.41, *P* < 0.001). Relative humidity also showed a significant positive main effect (*β* = 2.14, SE = 0.82, *P* = 0.01), but was modified by significant negative interactions in settlements (*β* =  − 1.90, SE = 0.94, *P* = 0.04) and wet peatlands (*β* =  − 2.00, SE = 0.81, *P* = 0.01). Precipitation showed a significant negative main effect (*β* =  − 1.84, SE = 0.92, *P* < 0.05), with no significant interactions in settlements (*β* =  − 0.47, SE = 1.43, *P* = 0.74) or wet peatlands (*β* = 1.73, SE = 0.90, *P* = 0.06), the latter marginally missing significance. Wet peatlands showed significantly higher abundance than the reference (*β* = 2.25, SE = 1.06, *P* = 0.03), whereas settlements did not differ significantly from it (*β* =  − 0.67, SE = 1.34, *P* = 0.62; post hoc results in Additional File 2, page 4). Marginal trends (Fig. 3B) for mean daily temperature were consistently positive across all habitat types (*β* = 0.47, 95% CI [0.24, 0.70]), reflecting the absence of a habitat interaction for this variable. Relative humidity trends, in contrast, varied by type: strongly positive in drained peatland (*β* = 0.39, 95% CI [0.10, 0.67]) but small in settlements (*β* = 0.04, 95% CI [− 0.20, 0.29]) and wet peatland (*β* = 0.03, 95% CI [− 0.12, 0.17]). Precipitation trends were negative across all types, more pronounced in drained peatland (*β* =  − 0.08, 95% CI [− 0.16, − 0.002]) and settlements (*β* =  − 0.10, 95% CI [− 0.21, 0.01]), but minimal in wet peatland (*β* =  − 0.005, 95% CI [− 0.04, 0.03]). The corresponding IRRs are presented in Additional file 2, Fig. 5B.

## Discussion

*Anopheles algeriensis* has been considered sympatric with *An. claviger* s.s. since its first detection in Germany nearly a century ago [33, 60]. While recent studies have revealed differences in structure, biogeochemistry, and vegetation of either species' preimaginal stages habitat in considerable detail, including in wetland habitats [32, 35, 74], neither species has been examined as epidemiologically relevant host-seeking adult females, nor in the context of peatland rewetting. Here, we close this gap by providing weather-contingent, habitat-related species distribution patterns.

### Factors shaping the ecological niche of *An. claviger* s.s.

*An. claviger* s.s. abundance was significantly higher in settlements than in either peatland type, indicating that anthropogenic landscapes offer more favourable developmental conditions. High sympatric abundance of *Culex* spp. at these sites indicates a predominance of permanent water bodies. This likely also explains outlier site RW-3, a rewetted former grassland with extensive permanent water and reed belts, the only site outside settlements with comparably high *An. claviger* s.s. abundance, which may account for the non-significant GLMM result between wet peatlands and settlements. Although consistent with the species' known preference for lentic, littoral ecotones [25, 60], shallow lake formation results from uncontrolled or unintended rewetting and is discouraged in rewetting practice guidelines [75, 76]. Nevertheless, this ambiguity highlights the ecological flexibility of *An. claviger* s.s.. The weather-contingency is evident in relative humidity interacting positively with settlements but negatively in both peatland types, indicating settlement populations benefit from rising humidity whereas peatland populations already operate near their optimal threshold. In drained peatlands, low vegetation height likely increased sun and wind exposure and reduced resting sites, conditions that probably outweighed humidity effects [77, 78]. Such ambivalent humidity responses are similarly reported for other European wetland mosquitoes and remain an open research question [79, 80]. A comparable positive interaction between minimum temperature and settlement type, without a significant main effect of mean daily temperature, suggests broad thermal adaptation across the study range, with warmer nights raising adult activity rather than population size [36, 81]. The weaker pattern in wet peatlands likely reflects their cooler microclimate, while drained peatlands may again reflect the adverse conditions outlined above [82]. These results corroborate earlier reports of *An. claviger* s.s. exploiting anthropogenic habitats such as artificial water bodies, drainage infrastructure, and animal shelters in rural settlement [7, 27, 40] and support the characterisation of this species as ecological generalist.

### Factors shaping the ecological niche of *An. algeriensis*

*Anopheles algeriensis* showed a contrasting pattern, with substantially higher abundance in wet peatlands than in settlements or drained peatlands. Co-occurrence with *Aedes* spp. indicates a more dynamic hydrological regime dominated by floodwater breeding sites in the preferred habitats. Within wet peatlands, abundance peaked at the least anthropogenically influenced sites. Even near natural sites with minimal management yielded comparatively low abundance, suggesting a narrower ecological niche than the wet peatland type discussed here may imply. Its near absence in settlements, where *An. claviger* s.s. thrives, points to an adverse effect of anthropogenic disturbance and indicates ecological segregation of both species along a gradient from natural to anthropogenic habitats. Weather effects also differed markedly from *An. claviger* s.s. Mean daily temperature had a strong, consistently positive effect across all habitats, unmodified by interaction, indicating a general bottom up constraint on population growth consistent with the species' known thermophily [19, 29, 36]. The positive effect of relative humidity was strongest in drained peatlands but attenuated in wet peatlands and settlements, suggesting that moisture availability constrains habitat use in drained peatlands specifically, whereas moisture is not limiting in wet peatlands and habitat suitability is inherently low in settlements [80]. The negative effect of precipitation, most pronounced in drained peatlands and settlements, may seem counterintuitive but may reflect a flushing or dilution effect on larval populations in hydrologically disturbed habitats [83], or a failure to generate suitable breeding sites outside wet peatlands combined with reduced flight activity [55, 84]. The near zero trend of precipitation in wet peatlands on the other hand suggests resilience of these habitats to rainfall-driven disturbance, likely attributable to the water retention capacity and structural complexity of wet peatlands, which attenuate hydrological fluctuations and flow, while dense reed vegetation may provide sheltered resting sites [85, 86]. This microclimatic buffering may also explain occasional mass developments of *An. algeriensis* well north of its core range [29]. Historical and recent records describe breeding sites in small, shaded water bodies of varying permanence within dense sedge (*Carex* spp.) or reed (*Phragmites* spp.) stands, supporting an association with tall graminoid vegetation [25, 32, 33]. Especially collection sites in Germany and neighbouring countries support a fen association of *An. algeriensis* in temperate Europe as they are consistently described as remote, undisturbed wetlands near lakes, ponds, and wet meadows [27, 28, 33] or even name specific peatlands such as the *Hinsbecker Bruch*, the site of the species' first national record [60], or the *Murnauer Moos*, which was recently confirmed as a persistent breeding site via 32 male specimens collected by Malaise trap in 2013 (Kuhlisch, pers. comm.) [29]. Hence, a concentration of records in fen-rich states such as Bavaria, Brandenburg, and Mecklenburg-Vorpommern may reflect genuine habitat preference, though sampling artefacts cannot be excluded. In any case, together with *Culiseta ochroptera*, *Ae. pionips*, and *Cx. martinii*, *An. algeriensis* is one of four rare mosquito species recorded associated with peatlands in Germany [87–89] which may indicate, that these habitats serve as refugia for rare species. In summary, our results indicate, that *An. algeriensis* occupies a comparatively narrow ecological niche in minimally disturbed wet peatlands defined by tall graminoid vegetation [34] with stabile microclimatic conditions [36, 90].

### Implications for peatland rewetting and management opportunities

Historical records and the present study link *An. algeriensis* to dense reed and sedge stands. Rewetting may induce helophytisation, a dominance shift towards tall wetland vegetation linked to altered water table dynamics and geochemistry [34]. Hence, although the landscape features analysed in this study did not allow to distinguish near-natural and rewetted peatlands, rewetting should in principle favour *An. algeriensis* [34]. *Anopheles claviger* s.s., by contrast, was associated with anthropogenic habitat structures and while certain permanent water bodies may be colonised, rewetting is unlikely to boost its abundance, unless unintended outcomes provide such water bodies [75, 76]. Consequently, minimally disturbed, helophyte-rich post-rewetting trajectories might be generally more advantageous for *An. algeriensis*, while excessive inundation may instead promote *An. claviger* s.s.. These findings may be used to develop vector management strategies. As most drained German peatlands are used agriculturally, rewetting often aims to establish paludiculture, the productive use of wet peatlands including reed harvesting or buffalo husbandry [91]. The resulting low vegetation structure increase sun and wind exposure while reducing resting sites, which may disadvantage or suppress both vectors [77, 78]. Paludiculture can also boost biodiversity [92], increasing predation on all life history stages [74], and if implemented as sustainable wetscape-use, may create barrier zones that reduce mosquito pressure near settlements [55, 93]. Furthermore, best practice approaches like slow rewetting and topsoil removal might limit pool formation and therefore breeding-site availability [94, 95]. Nevertheless, neither approach has been scientifically evaluated, thus, future research should investigate the effects of those management strategies on mosquito populations.

### Limitations of the study

Given the effective detection range of the trap systems used here [46], and the limited average foraging range of mosquitoes [55], trap data likely provide an ecologically meaningful representation of local populations. Nonetheless, the absence of larval sampling constitutes the main limitation of this study. Physicochemical parameters such as pH and salinity shape *Anopheles* spp. breeding sites, including those of the species examined here [35], and are themselves altered by peatland rewetting [34]. These parameters also influence competitive dominance between *Anopheles* larvae and other species, such as *Culex pipiens/torrentium* sympatric with *An. claviger* s.s. [96]. The presence of *Aedes cinereus/geminus* alongside *An. algeriensis*, however, may instead reflect a lack of sufficiently persistent breeding sites for *An. claviger* s.s. to establish [25], rather than physicochemical suitability, and may therefore not be linked to habitat types. Additional interspecific competition may arise not only from other mosquito species but also from controphic taxa [97]. Landscape and vegetation management further shape the composition of both mosquito and predator communities [74]: all those aspects are potentially influencing oviposition, species composition, and emergence in ways not necessarily captured by trap data. Furthermore, while flight capacity data are lacking for *An. algeriensis*, and only a single observation exists for *An. claviger* s.s., biotic and abiotic factors such as blood-host availability and resting site distribution may nonetheless drive dispersal beyond purely radial flight patterns as they are assumed in this study [55]. Future research should aim to connect these aspects, for instance through combined larval and adult sampling across a defined rewetting gradient, to clarify the mechanisms underlying the distribution patterns observed here.

### Larger context and perspective

The contrasting ecological profiles of the two vector species demonstrate the need to incorporate landscape context into mosquito species distribution models: climatic drivers, and the direction and magnitude of their effects, varied not only between species but, in several cases, among habitat types within species. Despite the limitations, these findings suggest that land-use change, including peatland rewetting, may alter local mosquito community composition and thereby affect pathogen transmission. However, despite altered abundance and distribution of *An. claviger* s.s. and *An. algeriensis*, the associated pathogen transmission risk, including for *Plasmodium* spp., is likely to remain low for these vectors [13]. Other vectors, however, may respond differently to rewetting and consequently constitute greater epidemiological concern [18]. The results indicate that large-scale climate–abundance relationships derived without accounting for landscape heterogeneity may misrepresent local dynamics and should be complemented by habitat-informed studies at ecologically relevant scales. Landscape-modulated climatic responses of mosquito vectors should be further investigated to inform risk assessment within a One Health framework. Consequently, vector surveillance must become an integral component of rewetting efforts.

## Conclusions

Adult *An. claviger* s.s. and *An. algeriensis* show distinct distribution patterns in peatland habitats, with the former thriving in anthropogenic landscapes and the latter restricted to helophyte-rich wetlands. As a consequence, rewetting trajectories that promote dense graminoid vegetation are likely to favour *An. algeriensis*, while the formation of permanent water bodies or continued anthropisation of peatlands may benefit *An. claviger* s.s. Paludiculture practices reducing vegetation height and structural complexity may suppress both species, enhance natural predation and may effectively buffer settlements from mosquito pressure. While pathogen transmission risk associated with *An. claviger* s.s. and *An. algeriensis* is likely to remain low despite rewetting efforts, incorporating habitat-informed vector surveillance into landscape restoration planning is essential for a robust One Health risk assessment of mosquito vectors in peatland rewetting practices.

## Supplementary Information

Additional file1 (XLSX 12 KB) Detailed description of sampling sites.

Additional file2 (DOCX 1716 KB) Additional information and results. Fig. 1) Maps of principal landscape characteristics. Fig. 2) Cluster dendrogram from which habitat types are derived. Fig. 3 & Fig4 Data distribution visualisation for both vector species. Post hoc results for both species’ GLMMs. Fig. 5) Dot and Whisker plot for the results of each species' model.

Additional file3 (XLSX 22 KB) Detailed results of exploratory analysis (both vectors).

Additional file4 (CSV 61 KB) Catch data of *Anopheles* vectors and all females of co-occurring mosquito species that were identified to species (complex) level.

## Abbreviations

*Ae.*: *Aedes*
*An.*: *Anopheles*
AOI: Area of interest
*CO1*: Cytochrome C oxidase unit 1
*Cs.*: *Culiseta*
*Cx.*: *Culex*
DLM: Digital landscape model
DWD: Deutscher Wetterdienst (German Weather Service)
GHG: Greenhouse gas
GLMM: Generalised linear mixed model
*ITS2*: Internal Transcribed Spacer 2
IRR: Incidence rate ratio
s.s.: Sensu stricto

## Declarations

### Availability of data and materials

All data generated or analysed during this study are included in this published article and its supplementary information files.
