# Supplementary material for: The rare and the common: habitat suitability and niche segregation of Anopheles algeriensis (Theobald, 1903) and Anopheles claviger s.s. (Meigen, 1804) in North-German peatlands is shaped by distinct landscape–weather interactions
Source: Parasit Vectors. 2026 Sep 23;19:410. doi: 10.1186/s13071-026-07612-w (PMC13602720; doi:10.1186/s13071-026-07612-w)
Supplement: Supplementary file 1 [file 13071_2026_7612_MOESM2_ESM.docx]

Figure 1: The three principal characteristics used to derive habitat types. Open Water and settlements handled as described in „Methods“, other features defined as follows: A) Vegetation directly renamed from the German DLM object type group (Objektartengruppe): „Vegetation“ and visually confirmed at sites. B) Soil status: Rewetting status of peat soils (44) extrapolated from representative water table measurement sites indicated by grey round crosses. Typology derived from water level classes in Hammerich et. al. 2022 (43), wet = 4+ and above, moist = 3+, drained = 2+ and below. C) Intensity derived from combination of DLM (see: Vegetation) and management practice: low intensity = grassland, mown once per year in autumn/winter; medium intensity = grassland mown several times per year or cattle pasture, no additional management; high intensity = cropland, conventional management including tilling and fertilizing. Information from local practitioners and visually confirmed at sites. The DLM is available as vector data from <https://www.laiv-mv.de/>. It is sampled with a scale of 1:10,000 and has a target scale of 1:25,000 with a point accuracy of ±3 m.Figure 2: Cluster Dendrogram constructed following the description in „Methods“ with the vegan package using Euclidean distance and wardsD2 linkage with the „vegdist“ and „hclust“ functions. Left habitat type = „wet peatlands“, centre habitat type = „drained peatlands“ and right habitat type = „settlements“.

Figure 3: Cullen and Frey distribution graph of Anopheles algeriensis constructed using the „descdist“-function in the fitdistrplus package

Figure 4: Cullen and Frey distribution graph of Anopheles claviger s.s. constructed using the „descdist“-function in the fitdistrplus package

Post-hoc analysis *An. claviger s.s.*:

Post hoc analysis using analysis of deviance (Type III ANOVA) confirmed that the interaction between habitat type and minimum temperature (*χ²* = 6.2, *df* = 2, *P* < 0.05) and the interaction between habitat type and relative humidity (*χ²* = 6.3, *df* = 2, *P* = 0.04) were significant predictors overall. The minimum temperature (*χ²* = 0.19, *df* = 1, *P* = 0.66) and relative humidity (*χ²* = 1.38, *df* = 1, *P* = 0.24) were not significant as individual predictors and neither was the habitat types as an individual main effect (*χ²* = 6.0, *df* = 2, *P* > 0.05).

Post-hoc analysis *An. algeriensis*:

Post hoc analysis with the Type III ANOVA confirmed that the mean temperature (*χ²* = 15.6, *df* = 1, *P* < 0.001), relative humidity (*χ²* = 6.8, *df* = 1, *P* = 0.009) and precipitation (*χ²* = 4.0, *df* = 1, *P* = 0.046) were significant individual predictors, as were habitat types (*χ²* = 8.2, *df* = 2, *P* = 0.02). The interaction between habitat type and relative humidity was a significant predictor overall (*χ²* = 6.2, *df* = 2, *P* < 0.05), whereas the interaction between habitat type and precipitation was not significant (*χ²* = 5.7, *df* = 2, *P* = 0.06).

Figure 5: Dot and Whisker plot visualizing the transformed incidence rate ratios (IRRs) with 95% CI for A) An. claviger s.s.and B) An. algeriensis including levels of significance (“*” = .05, “**” = .01, “***” = .001) constructed using the model_plot function of the sjPlot package.
